# Paralytic ileus in the neonate as a rare complication of maternal methadone treatment—a case report

**DOI:** 10.1093/omcr/omab004

**Published:** 2021-03-08

**Authors:** Joshua D Emery, Veronica M Samedi, William T Bingham

**Affiliations:** Department of Pediatrics, Neonatal Intensive Care Unit, Royal University Hospital, University of Saskatchewan, 103 Hospital Dr, Saskatoon, Saskatchewan, Canada

**Keywords:** paralytic ileus, neonate, maternal methadone

## Abstract

Narcotic bowel syndrome is defined as worsening abdominal bloating and cramping with chronic opiate use, leading to paralytic ileus. This syndrome is common yet underreported in adults. However, there is no current evidence of such conditions in the newborn after exposure *in utero* to high doses of opiates. Our patient was a female indigenous preterm infant born to a mother on a high dose of methadone. On admission at the age of 12 h, she was found to have significant gastric distension. Initial abdominal X-ray showed a large gastric bubble with little evidence of rectal gas. Malrotation was suspected and surgical intervention was discussed. However, repeat abdominal X-ray, ultrasound and upper Gastrointestinal series were found to be normal and without acute findings. Thus, surgery was avoided. The gastric distension resolved spontaneously. She never required opiate therapy for neonatal abstinence syndrome. Given the pattern of gas seen on the initial abdominal X-ray and its spontaneous resolution after removal of maternal methadone, we suspect this baby had neonatal narcotic bowel syndrome. This has never been reported in the literature and is a unique finding. Given the lack of current reports, further observations for this syndrome should be conducted.

## INTRODUCTION

Opioids are potent analgesic agents used to treat severe pain that is both acute and chronic in nature [1]. Common side effects include constipation and bowel dysfunction [1]. This is a result of action of opioids on receptors that line the gastrointestinal tract [1]. Chronic opiate use could lead to the bowel dysfunction, constipations or paralytic ileus [2]. In the pregnant population, illicit opioid use is found in approximately 0.1% of all pregnancies [3]. Currently, the recommended pharmacotherapy for opioid dependency in the antenatal period is methadone, which was found to diminish illicit use [3–5]. The most common side effect of maternal methadone and opiate use is neonatal abstinence syndrome (NAS), a set of signs and symptoms related to nervous system dysfunction that increases morbidity and prolongs NICU stay [3, 5]. In the available literature, we did not find reports describing early-onset gastrointestinal dysfunction in the neonates exposed to opiates and methadone *in utero*. In the given case report, we present a neonate with physical signs and radiologic evidence of ileus secondary to maternal methadone ingestion.

## CASE REPORT

This baby girl was born via home spontaneous vaginal delivery at approximately 32 weeks to a mother with no prenatal care or ultrasounds. She was HIV negative, and the remainder of serology results and GBS status were unknown. Subsequent postnatal screening was negative. The mother had a remote history of illicit drug use and was on maintenance therapy with methadone 100 mg/day throughout pregnancy. She was taking it as prescribed, which was confirmed by home visits. She had no other illicit substance use. She did intermittently smoke cigarettes and marijuana during the first trimester of pregnancy. She did not specify any alcohol use. Baby was born at home by spontaneous precipitous delivery and brought to a peripheral hospital by ambulance for further care. Her birth weight was 1392 g. Secondary to worsening respiratory distress, she was intubated and transferred to our tertiary care center.

**Figure 1:**
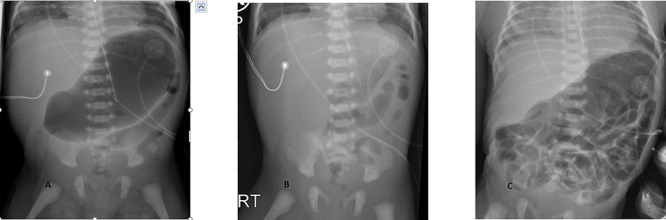
(**a**–**c**) Progression of the abdominal X-ray from an appearance of gastric outlet obstruction (a) to fairly decompressed (b) to a complete pattern of distension throughout the stomach, small and large bowels (c).

On admission, she was screened for infection and started on empiric antibiotics. Blood cultures were negative. Baby was clinically stable but was found to have abdominal distension and was nil per os. The initial abdominal X-ray (Fig. 1a) showed massive gastric distension with minimal gas pattern in the rest of the gut. There was no concern of duodenal atresia given the gas pattern; however, gastric outlet obstruction or malrotation could not be ruled out. Intermittent NG suctioning was then applied for gastric decompression and only air was aspirated. No vomiting or bilious aspirates were noted. An abdominal X-ray was repeated an hour later, showing the stomach had been deflated with air again being present in the distal transverse, descending and rectosigmoid colon (Fig. 1b). A further X-ray was done 2 h later showing reaccumulation of air in the stomach and bowel loops distended throughout the left abdomen (Fig. 1c).

**Figure 2:**
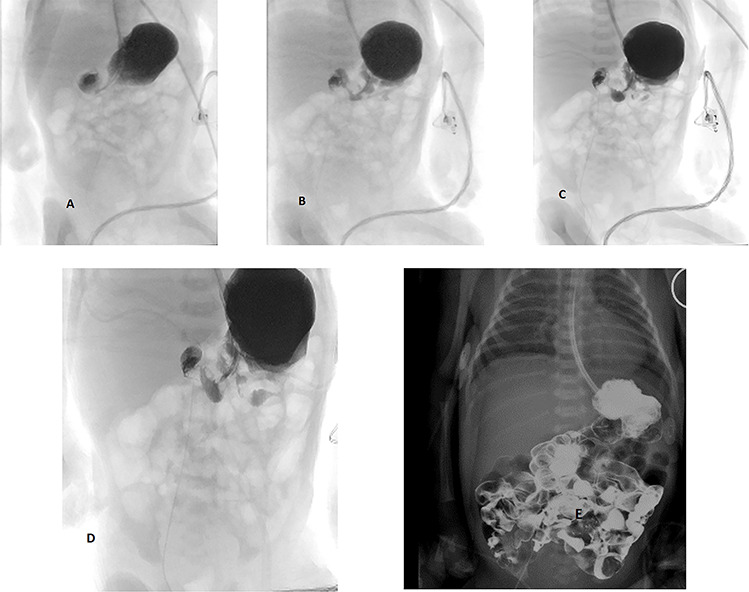
(**a**–**c**) Normal upper GI series; (**d**, **e**) normal upper GI series and final X-ray showing normal distribution of contrast throughout the small and large bowel.

Therefore, an abdominal ultrasound was conducted to rule out malrotation and assess the gut vasculature. The superior mesenteric artery and vein were found to be in normal orientation. Furthermore, pediatric surgery was promptly consulted after the ultrasound and saw no acute need for exploratory laparotomy. They recommended an upper Gastrointestinal (GI) series after 2 days of gastric decompression. In the meantime, the patient was hemodynamically stable. The upper GI series showed no acute obstruction or other significant findings (Fig. 2a–d). The abdominal distension resolved within 72 h of life at which time she passed meconium. Feeds were started with preterm formula at the same time and were tolerated well. No further abdominal foci was identified. She was successfully extubated on Day 7 of life and off respiratory support by Day 9 of life. Screening head ultrasounds and chest X-rays were normal. The infant had no clinical signs of NAS. Feeds were progressed over the subsequent 3 weeks on standard formula prior to discharge after a total of 6 weeks in NICU.

## DISCUSSION

For more than 30 years, methadone has been used as a first-line medication for opioid substitution therapy during pregnancy [5]. Despite the strong evidence supporting the use of methadone in pregnancy, methadone use is not without risk or side effects [5, 6]. Methadone does not only cross the placental barrier but is also retained by the placenta in a substantial amount [4]. There is not enough data describing fetal opioid elimination, however, placental inactivation of methadone is significantly lower than that of the maternal liver [6–8]. The elimination half-life of methadone in an opioid-tolerant patient is approximately 24 h; and in neonates, it could be as high as 40–55 h [6, 7]. The extended elimination half-life in neonates contributes to higher mg per kg-based dose [6].

There is well-documented evidence of delay of feeding onset and attaining full feeds in neonates with NAS or exposed to morphine in the NICU for analgesia [9, 10]. Fortunately, these effects are transient with no acquired GI complications [7, 8]. Our patient was off respiratory support in 9 days. Her infectious status was negative. However, abdominal distension persisted for 72 h. Her mother did not use other substances late in pregnancy other than methadone. Given that the neonate reported in this case study did not show signs of NAS, it seems less likely that we can contribute her clinical picture of ileus to her methadone exposure. However, the preterm neonates have lower rates of NAS compared to term babies [8], so she may not have displayed symptoms of withdrawal due to her prematurity. This association may well be due to the challenges of assessment of NAS in preterm infants and a lack of a validated scoring system specifically designed for this population [9]. It is important to consider maternal substance use when interpreting abdominal films and clinical signs of a surgical abdomen. These findings may help reduce the need for exploratory laparotomy in neonates presenting with a query of a surgical abdomen, if maternal substance use history is known.

Interestingly, there was a case report of an infant in Italy who experienced similar paralytic ileus after treatment with fentanyl for procedural sedation while ventilated [10]. Although the exposure was of a different nature, it is important nevertheless to know that any opioid derivative has the capacity to induce ileus in the neonate, whether prenatal or postnatal. Furthermore, another preterm infant whose mother was taking clonazepam, a benzodiazepine for epilepsy, was also born with concern of a paralytic ileus [10]. This adds further support to the argument that neurologically depressing agents use by a mother during pregnancy, such as opioids and benzodiazepines, must be considered when observing an infant with radiological and clinical signs of an obstructive pathology before advancing that neonate for laparotomy.

In conclusion, the given neonate was born premature after significant *in utero* exposure to methadone and was thought to have a malrotation on imaging. After ruling out a surgical cause of obstruction, the only factor that could be associated with such significant ileus was determined to be maternal methadone consumption. Given the pattern of gas seen on the initial abdominal X-ray and its spontaneous resolution after 72 h, we suspect this baby had paralytic ileus secondary to maternal methadone treatment. This has never been reported in the literature and is a unique finding. Hence, this correlation is a reminder to neonatology decision makers that maternal substance use may be a significant considering factor when sending a patient with small bowel obstruction to the operating theater. Given the lack of current reports, further surveillance for this phenomenon should be conducted.

## CONFLICT OF INTEREST

None declared.

## FUNDING

No sources of funding were used.

## ETHICS APPROVAL

Ethics approval for said case report was waived. Parental consent was gained prior to creating this manuscript.

## CONSENT

All images and information presented about the given minor were used with informed consent from the individual’s legal guardian. All images were anonymized.

## GUARANTOR

Veronica Mugarab Samedi is a guarantor for this publication.
